# Caveolae-mediated entry of *Salmonella typhimurium* into senescent nonphagocytotic host cells

**DOI:** 10.1111/j.1474-9726.2010.00554.x

**Published:** 2010-04

**Authors:** Jae Sung Lim, Hyon E Choy, Sang Chul Park, Jung Min Han, Ik-Soon Jang, Kyung A Cho

**Affiliations:** 1 Department of Biochemistry, Chonnam National University Medical School5 Hakdong, Gwangju, Korea; 2 Research Institute of Medical Sciences, Chonnam National University Medical School5 Hakdong, Gwangju, Korea; 3 Department of Microbiology, Chonnam National University Medical School5 Hakdong, Gwangju, Korea; 4 Department of Biochemistry and Molecular Biology, Aging and Apoptosis Research Center, Seoul National University College of MedicineYongondong, Seoul, Korea; 5 Research Institute of Pharmaceutical Sciences, College of Pharmacy, Seoul National UniversitySeoul, Korea; 6 Basic Science InstituteDaejun, Korea

**Keywords:** senescence, caveolae, caveolin-1, *S. typhimurium*, infection, endocytosis

## Abstract

Elderly individuals have an increased susceptibility to microbial infections because of age-related anatomical, physiological, and environmental factors. However, the mechanism of aging-dependent susceptibility to infection is not fully understood. Here, we found that caveolae-dependent endocytosis is elevated in senescent cells. Thus, we focused on the implications of caveolae-dependent endocytosis using *Salmonella typhimurium*, which causes a variety of diseases in humans and animals by invading the eukaryotic host cell. *Salmonella* invasion increased in nonphagocytotic senescent host cells in which caveolin-1 was also increased. When caveolae structures were disrupted by methyl-β-cyclodextrin or siRNA of caveolin-1 in the senescent cells, *Salmonellae* invasion was reduced markedly compared to that in nonsenescent cells. In contrast, the over-expression of caveolin-1 led to increased *Salmonellae* invasion in nonsenescent cells. Moreover, in aged mice, caveolin-1 was found to be highly expressed in Peyer’s patch and spleen, which are targets for infection by *Salmonella*e. These results suggest that high levels of caveolae and caveolin-1 in senescent host cells might be related to the increased susceptibility of elderly individuals to microbial infections.

## Introduction

Caveolae are flask-shaped invaginations in the plasma membrane on the surface of endothelial cells (Palade, 1953; Yamada, 1955). It has been suggested that caveolae mediate the extensive transcellular shuttling of serum proteins from the blood stream into tissues across the endothelial cell layer (Simionescu *et al.*, 1975; Ghitescu & Bendayan, 1992; Schnitzer *et al.*, 1994). Caveolae present on various types of cells consist of cholesterol and sphingolipid-rich microdomains of the plasma membrane (Brown & London, 1998; Simons & Toomre, 2000), in which many diverse signaling molecules and membrane transporters are concentrated. This organelle mediates the internalization of sphingolipids and sphingolipid-binding toxins, glycosylphisphatidylinositol (GPI)-anchored proteins, and growth hormones (Nichols & Lippincott-Schwartz, 2001).

Caveolae are composed mainly of caveolin proteins. Caveolins are a family of a 21–24 kDa integral membrane proteins consisting of caveolin-1, -2, and -3. Caveolin-1 and -2 are expressed together and form a hetero-oligomer in the plasma membrane of many cell types (Smart *et al.*, 1999), whereas caveolin-3 is expressed only in muscle tissue (Tang *et al.*, 1996). Caveolin-1 is a scaffolding protein within the caveolae membrane and interacts with various signaling proteins such as epidermal growth factor (EGF) receptor, G-proteins, Src-like kinases, Ha-Ras, protein kinase C, endothelial nitric-oxide synthase, and integrin (Li *et al.*, 1996; Garcia-Cardena *et al.*, 1997; Razani *et al.*, 1999; Smart *et al.*, 1999). Previously, we reported that the expressions of caveolin-1 and caveolae structures are higher in senescent cells, leading to the suppression of EGF-dependent growth activity and to the induction of senescence-associated morphological changes (Park *et al.*, 2000; Cho *et al.*, 2003, 2004).

Caveolae are involved in the entry of several species of viruses, parasites, and bacterial toxins. In the case of human immunodeficiency virus (HIV), the endocytosed microbial cargo is transported directly across the cell (Abrami *et al.*, 2000). The entry of simian virus 40 (SV40) into host cells is mediated by major histocompatibility complex (MHC) class I molecules, after which the virus is transported by caveolae to the endoplasmic reticulum (ER) (Atwood & Norkin, 1989; Anderson *et al.*, 1996). Cholera toxin (CT) binds first to the plasma membrane ganglioside (GM1), which is highly concentrated in caveolae, and subsequently this complex is transported into the ER through caveolae (Parton *et al.*, 1994; Lencer *et al.*, 1999). Shin *et al.* also provided evidence suggesting the possibility of caveolae-mediated entry of bacteria using FimH-expressing *Escherichia coli*. They showed that CD48, a receptor for FimH-expressing bacteria, is localized within caveolae and FimH-expressing *E. coli* are also co-localized with caveolae via interaction with CD48 in mast cells (Shin *et al.*, 2000).

Because caveolae have been implicated in the entry of microbial pathogens, we examined their role in the entry of *Salmonella typhimurium* into host cells as this bacterium enters the eukaryotic host cell and replicates therein. The entry of *Salmonella* into animal host cells requires complex, syringe-like macromolecular structures termed type III secretion systems (TTSS) encoded by *Salmonella* Pathogenecity Island, SPI (Ohl & Miller, 2001). TTSS translocates a number of effector proteins including SopE, SopE2, and SopB, also encoded by SPI (Galan, 2001; Zhou & Galan, 2001). The SopE mimics a guanine nucleotide exchange factor that interacts with Rac1 and Cdc42, promoting actin cytoskeletal reorganization and mediating the internalization into host cells.

Therefore, in this work, we have assumed that the high level of caveolin-1 in aged cells might contribute to the higher susceptibility to microbial infection seen in older individuals. In addition, we report that the entry of *Salmonella* into host cells through caveolae-dependent endocytosis could be up-regulated in the senescent cells, probably resulting in the higher microbial susceptibility of older people.

## Results

### Transport-related genes are affected by the level of caveolin-1

**Fig. 1:**
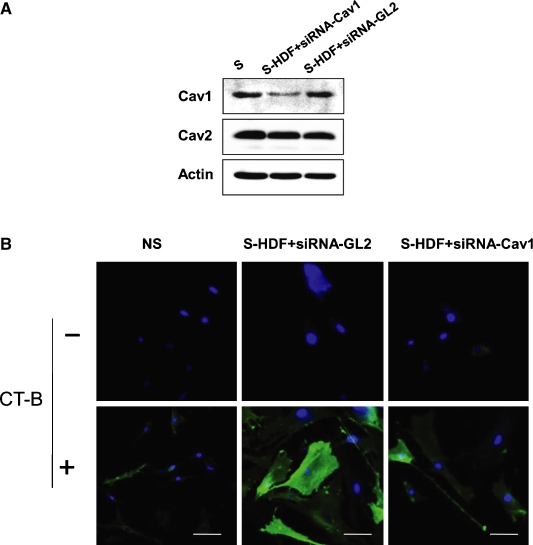
Caveolae-dependent uptake of fluorescent cholera toxin (CT) B subunits. Senescent human diploid fibroblasts were transfected with siRNA targeting of caveolin-1 (Cav1) or firefly luciferase (GL2), as a control. (A) Down-regulation of caveolin-1 expression was confirmed by Western blotting with anti-caveolin-1, -caveolin-2, -caveolin-3, and -actin antibody. (B) Cells were treated with fluorescent CT-B subunits (green) for 30 min and then fixed with 4% paraformaldehyde solution. Fixed cells were stained with DAPI (blue) and analyzed by confocal microscopy. Upper panel shows CT-B untreated cells, and lower panel shows CT-B-treated cells. Data are representative of > 100 cells observed in at least five independent experiments. Scale bar, 10 μm.

Previously, we reported that caveolin-1 increases in senescent human diploid fibroblasts (HDFs) and plays an important role in senescence-associated growth responses and morphological changes (Park *et al.*, 2000; Cho *et al.*, 2003, 2004). We speculated that the level of caveolin-1 alters the expression of certain genes, presumably those implicated in cellular senescence. Microarray analysis showed differences between the expression profiles of senescent HDFs and those in which the expression of specific siRNAs was reduced by caveolin-1. After confirming the effect of siRNA targeting to caveolin-1 (Fig. 1A; Park *et al.*, 2000; Cho *et al.*, 2003, 2004), we isolated mRNA from senescent and control siRNA-treated senescent or caveolin-1 siRNA-treated senescent cells and performed microarray analysis. Using a microarray analysis chip composed of 8000 human EST clones, we found 65 genes whose expressions were altered more than 2-fold by treatment with siRNA targeting caveolin-1, compared to control siRNA (Table 1). Of these, 23 genes were grouped into three classes of known genes: those involved in the cell cycle, cell growth and maintenance, and signal transduction. The remainder consisted of genes of unknown function. Among the genes involved in cell growth and maintenance, several transport-related genes were notably reduced, including those involved in iron transport (ATP2B1, SLC26A11), lipid transport (APOE, HDLBP), protein transport (CHM, NPC1), and endocytosis (SLC6A1, VAMP3), suggesting that the level of caveolin-1 expression primarily affected transport-related genes. We speculated that in the senescent cell, these transport-related genes are elevated in accordance with increased expression of caveolin-1, although it is unlikely that caveolin-1 would activate these genes directly.

**Table 1:** Caveolin-1 modulates transport-related genes

Group	Fold induction	Gene	Functions
Apoptosis	2.4	STK17B	Induction of apoptosis, protein amino acid phosphorylation
Cell cycle	2.0	TOP1	Cell growth and/or maintenance
	2.0	SMC5L1	Chromosome segregation
	−3.8	CENPE	Mitotic metaphase plate congression, DNA replication and chromosome cycle
	−2.0	PFDN1	Cell cycle
	−2.8	CENPB	Centromere/kinetochore complex maturation
Cell growth and maintenance	−2.1	ATP2B1	Cation transport, calcium ion transport
	2.6	MPHOSPH10	rRNA processing
	−2.2	CHM	C-terminal protein geranylgeranylation, intracellular protein transport
	−3.4	SLC6A1	Synaptic transmission, neurotransmitter transport
	−2.4	SLC26A11	Sulfate transport
	−2.8	APOE	Lipid transport, cholesterol metabolism
	−4.0	NPC1	Intracellular protein transport, cholesterol transport
	−2.0	VAMP3	Nonselective vesicle docking, membrane fusion
	−2.8	HDLBP	Lipid transport
	−2.0	IGFBP5	Signal transduction, regulation of cell growth
	−2.2	ACTN4	Invasive growth, cell motility
	−2.4	PFN2	Regulation of actin polymerization and/or depolymerization
	−2.6	TGFBR2	TGFβ ligand binding to type II receptor, positive regulation of cell proliferation
Signal transduction	−2.0	XPR1	Pathogenesis
	−2.0	ACCN3	Sensory perception, small molecule transport, signal transduction
	−2.4	RASAL2	Signal transduction
	−2.4	ELK2	Signal transduction, regulation of transcription from PolII promoter
Transcription	−2.4	LOC90233	Regulation of transcription, DNA-dependent

Subsequently, we examined the extent of endocytosis by senescent HDF by determining the uptake of fluorescein isothiocyanate (FITC)-conjugated CT B subunits (CT-B; Fig. 1B). CT-B is taken up by animal cells through caveolae-dependent endocytosis (Parton *et al.*, 1994). The uptake of FITC-conjugated CT-B was detected in senescent HDFs compared to nonsenescent HDFs. We also examined the uptake of CT-B in senescent HDFs in which caveoline-1 was down-regulated by specific siRNA and found that the uptake of CT-B was reduced to the level of nonsenescent HDFs. Taken together, these findings suggest that elevated caveolin-1 in senescent cells would be responsible for the increased transport-related gene expression, including that involved in endocytosis, which results in increased caveolae-dependent endocytosis.

### *Salmonella* entry increase in senescent host cells over-expressing caveolin-1

**Fig. 2:**
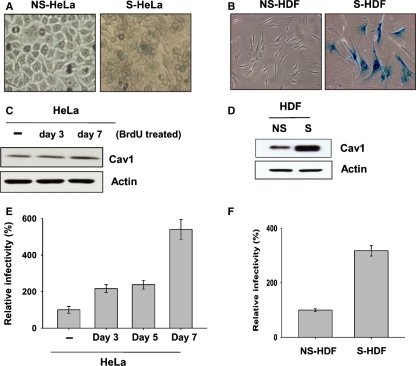
Increased *Salmonella* infection to senescent nonphagocytotic host cells Senescent cells were confirmed by senescence-associated β-galactosidase staining. (A) Senescent HeLa cells (S-HeLa) were induced with 5-bromo-2-deoxyuridine (BrDU) treatment over 7 days. Nonsenescent HeLa (NS-HeLa) and S-HeLa cells were stained with senescence-associated β-galactosidase staining. (B) Senescent human diploid fibroblasts (HDFs) were also confirmed by senescence-associated β-galactosidase staining. The expression levels of caveolin-1 (cav-1) in BrDU-treated S-HeLa (C) and nonsenescent (NS) and senescent (S) HDFs (D) were analyzed by Western blotting with anti-caveolin-1 (top) or anti-actin (bottom) antibodies. Data are representative of at least five independent experiments. *Salmonella* invasion of BrDU-treated senescent HeLa cells (E) or senescent HDFs (F) was quantified as described in Methods. Invasion is expressed as a percentage of wild-type bacteria internalized by control cells after 60 min. Data are the means (±SD) of three independent filters, and experiments were performed at least five times.

Because caveolae have been implicated in the endocytosis of microbial pathogens into host cells (Shin & Abraham, 2001; Pelkmans & Helenius, 2002; Laude & Prior, 2004), we investigated whether microbial invasion into senescent host cells would be elevated because of increased caveolin-1 expression using *S. typhimurium.* It is well known that *Salmonella* entry into nonphagocytotic intestinal epithelial cells is an essential early event in pathogenesis (Finlay & Falkow, 1997), a process that can be approximated *in vitro* using cultured cells. HeLa cells originating from epithelial cells have been used as representative models for the study of *Salmonella* invasion and virulence (Steele-Mortimer *et al.*, 2002). Thus, we first attempted to induce the senescent phenotype in HeLa cells using 5-bromo-2-deoxyuridine (BrDU) treatment. BrDU induces the senescent phenotype through its incorporation into DNA, which produces damage in cancer cells and results in phenotypic changes that resemble senescence, including reduced proliferation (Masterson & O’Dea, 2007). After 7 days, the HeLa cells treated with BrDU started to exhibit the senescent phenotype, as revealed by β-Gal staining, although the phenotype was slightly less pronounced than in naturally senescent HDFs (Fig. 2A,B). Subsequently, we examined the level of caveolin-1 expression in BrDU-treated HeLa cells with young and naturally senescent HDFs using a specific antibody. We found that caveolin-1 increased, in a time-dependent manner, to about 3-fold on Day 7 (Fig. 2C). A direct comparison revealed a notable increase in caveolin-1 expression in senescent HDFs (Fig. 2D). Next, we determined the extent of *Salmonella* invasion using nonsenescent and senescent HeLa cells (Fig. 2E). *Salmonella* invasion increased in BrDU-treated HeLa cells in a time-dependent manner; a gradual increase was observed up to 7 days (∼5.5-fold). Similarly, *Salmonella* invasion was elevated in senescent HDFs by approximately 3.5-fold compared to that in nonsenescent HDFs (Fig. 2F).

**Fig. 3:**
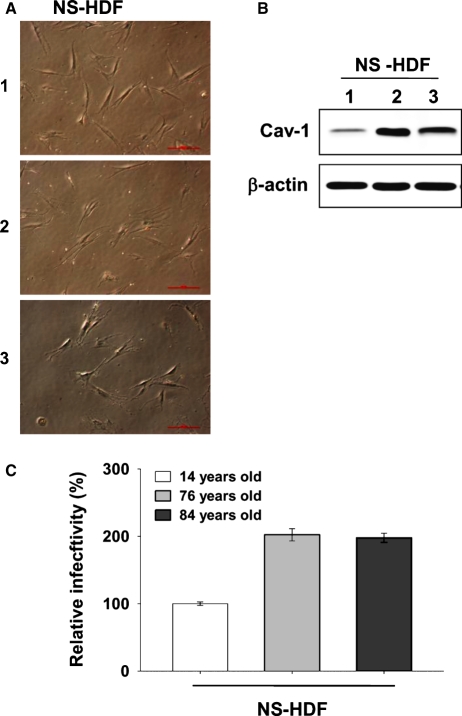
Increase of *Salmonella* infection of nonsenescent fibroblasts with age. Primary fibroblasts were isolated from donors aged 14, 76, or 84 years old. (A) Cells were stained with senescence-associated β-galactosidase staining solution. (B) Expression levels of caveolin-1 (cav-1) in nonsenescent (NS) cells were analyzed by Western blotting with anti-caveolin-1 (top) or anti-actin (bottom) antibodies. (C) *Salmonella* invasion to primary fibroblasts was quantified and expressed as a percentage of wild-type bacteria internalized by control cells after 60 min. 1: 14 years old; 2: 76 years old; 3: 84 years old. Data are the means (±SD) of three independent filters, and experiments were performed at least five times.

We examined the extent of *Salmonella* invasion in isolated primary human fibroblasts from young (14 years) and elderly donors (75, 84 years). Although primary fibroblasts from elderly donors did not show senescent phenotypes (Fig. 3A), *Salmonella* invasion was increased 2-fold in these fibroblasts compared to the fibroblasts from young donors (Fig. 3C). Interestingly, caveolin-1 was also highly expressed in primary fibroblasts from elderly donors (Fig. 3B).

*Salmonella* infected senescent and primary cells from elderly donors more readily than nonsenescent and primary cells from young donors, presumably because of the increased expression of caveolin-1. This suggests that the entry of *Salmonella* is not completely random but is mediated through caveolae.

### Caveolae mediate *Salmonella* invasion into host cells

**Fig. 4:**
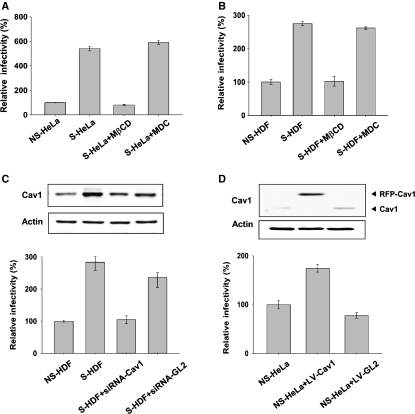
Implication of caveolae in *Salmonella* invasion of senescent host cells. Nonsenescent (NS) and senescent (S) HeLa cells (A) or human diploid fibroblasts (HDFs) (B) were treated with methyl-β-cyclodextrin (MβCD) or monodansylcadaverine (MDC). *Salmonella* infection of these cells was quantified as described. (C) Senescent HDFs were infected with lentivirus carrying the siRNA of caveolin-1 or firefly luciferase (GL2), as a control. The protein level was confirmed by Western blotting. *Salmonella* invasion was quantified after disruption of caveolae by siRNA in senescent HDFs. (D) To induce caveolin-1 in HeLa cells, we constructed a lentivirus carrying the caveolin-1 gene. *Salmonella* invasion was quantified in the HeLa cells that over-expressed caveolin-1 from the lentivirus. Invasion is expressed as a percentage of wild-type bacteria internalized by control cells after 60 min. Data are the means (±SD) of at least five independent filters, and experiments were performed at least three times. siRNA-CAV1, lentivirus carrying the siRNA of caveolin-1; siRNA-GL2, lentivirus carrying siRNA of GL2; LV-CAV-1, lentivirus carrying cavelin-1; LV-GL2, lentivirus carrying GL2.

We further examined the role of caveolae in *Salmonella* invasion by treating host cells with methyl-β-cyclodextrin (MβCD) to inhibit caveolae-dependent endocytosis (Yancey *et al.*, 1996) or with monodansylcadaverin (MDC) to inhibit clathrin-dependent endocytosis (Ray & Samanta, 1996). Senescent HeLa cells exhibited ∼5.5-fold higher *Salmonella* invasion compared to nonsenescent HeLa cells (Fig. 4A). This increase was abolished when the senescent HeLa cells were treated with MβCD, but not when they were treated with MDC. Senescent HDFs were invaded by *Salmonella* approximately three times more readily than were nonsenescent HDFs (Fig. 4B). Again, the higher infection rate was abolished when senescent HDFs were treated with MβCD, but not with MDC. These results suggested that caveolae-dependent endocytosis is indeed implicated in the *Salmonella* infection of senescent host cells.

Subsequently, we treated senescent HDFs with siRNA targeting caveolin-1 and determined the level of *Salmonellae* invasion. The extent of *Salmonella* invasion in siRNA-treated senescent cells was reduced to the level seen in nonsenescent HDFs (Fig. 4C). Conversely, increased expression of caveolin-1 in nonsenescent HeLa cells by infection with lentivirus carrying caveolin-1 led to an approximately 1.8-fold increase in entry by *Salmonella* (Fig. 4D). We concluded that *Salmonella* enter senescent host cells through caveolae-dependent endocytosis.

### Elevated expression of caveolin-1 in old mice

**Fig. 5:**
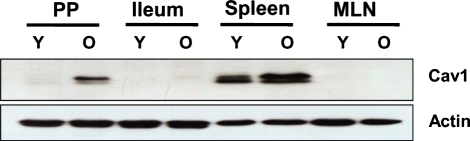
Differential expression of caveolin-1 in young and old organs. Organs were isolated from ‘young’ (2 months old, Y) and ‘old’ (25 months old, O) mice and analyzed for the expression of caveolin-1 by Western blot using anti-caveolin-1 antibody and anti-actin antibody. Data are representative of at least five individually analyzed mice. PP, Peyer’s patch; MLN, mesenteric lymph node.

It is known that *Salmonellae* infect animals through specialized M cells in the follicle-associated epithelium of intestinal Peyer’s patches (PP), which serve as portals for diverse particulates (Kerneis *et al.*, 1997; Sansonetti & Phalipon, 1999). Thus, we examined the expression of caveolin-1 in PP, spleen, mesenteric lymph node (MLN), and ileum in young (2 months old) and old (24 months old) mice, using a specific antibody. The level of caveolin-1 in PP and spleen was elevated significantly in the old mice, whereas caveolin-1 was virtually undetected in ileum and MLN in both young and old mice (Fig. 5A).

This suggests that the increased susceptibility of aged hosts to microbial infection could be attributable to the elevated expression of caveolin-1 and the subsequent increase in caveolae in PP and spleen.

## Discussion

In this study, we demonstrated that the level of caveolin-1 expression affects the expression of transport-related genes (Table 1). The elevated expression of transport-related genes in senescent HDFs was reduced by the down-regulation of caveolin-1 to the level observed in young HDFs. Consistently, significantly more FITC-conjugated CT-B was taken up by caveolae in the senescent cells than in the young cells, and this was reduced by the down-regulation of caveolin-1 to the level observed in the young cells (Fig. 1B). Although the underlying mechanism of the regulation of transport-related genes by caveolin-1 has yet to be elucidated, caveolin-1 expression appears directly related to molecule transport.

Most intracellular microbes enter host cells through clathrin-dependent endocytosis, an intracellular pathway in which lysosome fusion with internalized vesicles leads to the degradation of their contents (Veiga & Cossart, 2006). The microbes that enter host cells through this classical clathrin-dependent endocytosis must avoid degradation by the endosome–lysosome pathway, either by escaping from their endocytic vacuoles (phagosomes) into the cytoplasm before lysosomes fuse with phagosomes, or by actively neutralizing the microbicidal agents inside the phagosomes after fusion (Alpuche Aranda *et al.*, 1992). Unlike clathrin-dependent endocytosis, the caveolae-dependent endocytic vesicles do not fuse with lysosomes (Shin & Abraham, 2001); thus, microbes can readily avoid intracellular degradation. Various microbes use caveolae as a pathway for escaping degradation. The caveolae levels change dramatically with cellular conditions or functions. Accordingly, the extent of caveolae-dependent endocytosis of pathogenic microbes can also vary depending on the physiological status of the target organ. Although *Salmonella* entry into animal cells has been considered random, we have found that it occurs through caveolae-dependent endocytosis, which increases with animal age.

It is well known that elderly individuals have an increased susceptibility to infections. Here, we implemented a model to study age-related microbial infection using *Salmonella* that invaded host cells. We showed that the extent of *Salmonella* invasion was higher in two types of cellular aging models: HDFs and HeLa cells (Fig. 2E,F), and treatment with MβCD, an inhibitor of caveolae-dependent endocytosis reduced this to the level seen in young cells (Fig. 4A,B). Because MβCD depletes cholesterol in the cell membrane, whole cholesterol-rich membrane domains, including both lipid rafts and caveolae, are affected by treatment with MβCD (Yancey *et al.*, 1996; Xia *et al.*, 2004). Lipid rafts and caveolae are putative membrane microdomains with compositions different from the surrounding regions of the membrane. Both are enriched in cholesterol, glycosphingolipids, shpingomyelin, and GPI-linked proteins. Caveolae are a subset of lipid rafts characterized by flask-/omega-shaped membrane invaginations with caveolin proteins. To distinguish the role of caveolae and lipid rafts, we treated the cells with specific siRNA for caveolin-1, which specifically disrupted caveolae structures in senescent HDFs (Cho *et al.*, 2003). The degree of *Salmonella* entry in siRNA-treated senescent HDFs was reduced to the level observed in the young HDFs (Fig. 4C). In contrast, the over-expression of caveolin-1 in HeLa cells led to increased *Salmonella* entry (Fig. 4D). Taken together, these results suggest that caveolae are directly implicated in *Salmonella* infection.

Once *Salmonella* are ingested by the host animal, they travel through the gastrointestinal tract until they encounter M cells of the PP, through which they traverse the underlying tissue and then infect macrophages (Jensen *et al.*, 1998; Sansonetti & Phalipon, 1999). *Salmonella* not only survive but proliferate in macrophages and spread to other organs including liver and spleen. We observed that caveolin-1 expression was higher in the PP and spleen of old mice (Fig. 5). Taken together, these results provide the first evidence that elevated caveolin-1 and caveolae may be related to the age-dependent increase in susceptibility to microbial infection, although further study is needed to elucidate the underlying mechanism of caveolae-dependent *Salmonella* entry.

## Experimental procedures

### Reagents

Monoclonal anti-caveolin-1 antibody (C43420) was purchased from Transduction Laboratories (San Jose, CA, USA). Secondary horseradish peroxidase-conjugated anti-rabbit and anti-mouse antibodies were purchased from Jackson Immunochemicals (West Grove, PA, USA). Other biochemical reagents were purchased from Sigma Chemical (St Louis, MO, USA) or Life Technologies (Carlsbad, CA, USA).

### Cellular senescence model

HDFs were isolated from the foreskin of a 6-year-old boy. HDFs were maintained in 10-cm plates in Dulbecco’s modified Eagle’s medium (DMEM) supplemented with 10% fetal bovine serum (FBS) and 1% antibiotics. HDFs were subcultured at a ratio of 1:4. We defined young cells as those resulting from fewer than 25 population doublings and senescent cells from more than 60 population doublings. HeLa cells were induced senescent phenotypes, resulting from BrDU treatment over 7 days, as described by Michishita *et al.* (1999). Senescent cells were confirmed by their delayed population-doubling times and through a senescence-associated β-galactosidase activity assay, as described by Dimri *et al.* (1995). After reaching a semi-confluent state, senescence-associated β-galactosidase activity (pH 6.0) was examined. Briefly, cells were washed with phosphate-buffered saline (PBS) and then fixed with 2% paraformaldehyde containing 0.2% glutaraldehyde in PBS for 5 min at room temperature. After washing with PBS, cells were incubated with β-galactosidase reagent [1 mg mL^−1^ X-gal, 40 mm citric acid/sodium phosphate buffer (pH 6.0), 5 mm potassium ferrocyanide/potassium ferricyanide, 150 mm NaCl, 2 mm MgCl_2_] for 4 h at 37°C.

To study human aging, primary fibroblasts were obtained from buttock skin samples of young (14 years old) and elderly (76 and 84 years old) male donors. These cells were provided by Dr J. H. Chung (The Seoul National University College of Medicine).

### Animals

C57BL/6J mice were obtained from the Jackson Laboratory and maintained on a standard diet with food and water *ad libitum* in an animal facility and were used in accordance with the guidelines of the Institutional Animal Care and Use Committee of Chonnam National University Medical School. Old mice were maintained for more than 24 months; tumor-free female mice were used for this experiment. Two 1-month-old female mice were used as young controls.

### RNA interference and transfection

A synthetic siRNA duplex corresponding to the caveolin-1 mRNA sequence (5′-AACCAGAAGGGACACACAGUU-3′) was used to inhibit caveolin-1 protein expression (Cho *et al.*, 2003). A synthetic siRNA duplex corresponding to firefly luciferase (GL2) mRNA (5′-AACGUACGCGGAAUACUUCGA-3′) was used as a negative control. The siRNA duplexes were purchased from Dharmacon Research (Lafayette, CO, USA). The cells were grown to 50% confluence in 100-mm dishes and transfected with annealed siRNA using OligofectAMINE (Invitrogen, Carlsbad, CA, USA) for 6 h, as described by the manufacturer. The cells were then transfected with 0.5 nmole (7 μg) siRNA and Oligofectamine™ reagent in serum-free DMEM and incubated for 4 h at 37°C in a CO_2_ incubator. Following incubation, the cells were supplied with DMEM containing 10% FBS and harvested 48 or 72 h later for further analysis.

### cDNA microarray analysis

DNA microarray chips composed of 8000 human EST clones were provided by Digital Genomics Inc. (Seoul, Republic of Korea). RNA samples from senescent HDFs were used to make cDNAs labeled with Cy3, which served as reference probes. RNA samples from siRNA-GL transfected senescent and siRNA-CAV transfected senescent HDFs were used to make cDNA labeled with Cy5. Microarray experiments were performed according to the manufacturer’s standard protocol. Normalized data were obtained by the method of intensity/location-dependent normalization (Yang *et al.*, 2002). We compiled the genes from the normalized data that displayed 2-fold changes and ruled out the genes that changed after treatment with siRNA-GL in senescent HDFs.

### CT uptake assay

Cells were cultured on coverslips in 24-well plates and treated with CT-B conjugated with FITC (CT-B–FITC) for 30 min at 4°C. The cells were washed with cold DMEM and incubated at 37°C for 30 min. The cells were processed for immunocytochemistry as described previously (Lim *et al.*, 2000). Cells were fixed with 4% paraformaldehyde in PBS for 60 min and then mounted for detection by confocal microscopy.

### *Salmonella* invasion assay

Bacterial invasion assays were performed as described previously (Lee *et al.*, 1992). Overnight cultures of *S. typhimurium* were grown at 37°C in LB medium. *Salmonella typhimurium* were inoculated into fresh cultures and grown for 4 h at 37°C and then resuspended at the appropriate dilution in cell culture medium for infection of cell monolayers (HeLa multiplicity of infection (MOI) 1:10, HDF MOI 1:100) for 30 min. Cells were seeded (HDF, 1 × 10^4^ cells; HeLa, 1 × 10^5^ cells) in a 24-well dish and grown in DMEM with 10% FBS at 37°C in a 5% CO_2_ incubator. Infected cells were washed three times with PBS (pH 7.4). Then, DMEM containing gentamicin (10 μg mL^−1^; Sigma Chemical) was added, and the mixtures were incubated for 30 min. Intracellular bacteria were harvested by extraction with lysis buffer (0.05% Triton X-100 in PBS, pH 7.4) in triplicate for colony counting on brain–heart infusion agar plates.

### Inhibition of endocytosis

Senescent HDFs were seeded (1 × 10^4^) in 24-well plates and treated with 1% MβCD (Sigma Chemical) to disrupt cholesterol-rich membrane domains for 45 min in serum-free medium at 37°C in a CO_2_ incubator. Then, the cells were washed with PBS (pH 7.4) and used immediately for *S. typhimurium* invasion assays. Senescent HDFs were seeded (1 × 10^4^) in a 24-well plate and treated with 1% MDC (Sigma Chemical), to inhibit clathrin-coated pit formation for 30 min in serum-free medium at 37°C in a CO_2_ incubator. Then, the cells were washed with PBS (pH 7.4) and immediately used for *S. typhimurium* invasion assays.

### Infection of lentivirus

Lentivirus expressing full-length caveolin-1 gene was manufactured by Macrogen Co. (Seoul, Korea). Senescent HDFs were seeded (4 × 10^4^) in a 6-well plate and cultured until 60–70% confluent. The cells were infected with 1 mL of lentivirus for 8 h. After incubation, the cells were supplied with growth medium containing 10% FBS and harvested 48 or 72 h later for further assay.

### Western blot analysis

Peyer’s patches, ileum, MLN, and spleen were isolated from young (6–7 weeks old) and old (23–24 months old) mice. Tissues were homogenized in lysis buffer [50 mm Tris–HCl, pH7.5, 150 mm NaCl, 1 mm EDTA, 60 mm octyl β-d-glucopyranoside, 1 mm phenylmethysulfonyl fluoride, protease inhibitor cocktail (1:500; Sigma Chemical), 50 mm NaF, and 1 mm Na_3_VO_4_] with polytron homogenizers and then sonicated briefly. Protein samples were separated using sodium dodecyl sulfate polyacrylamide gel electrophoresis and transferred onto polyvinylidene fluoride transfer membrane (Pall Co., Pensacola, FL, USA). The membranes were incubated with a primary antibody overnight in a cold room, incubated with a peroxidase-conjugated anti-mouse or anti-rabbit secondary antibody for 1 h at room temperature and then visualized using an enhanced chemiluminescence detection kit (Amersham ECL kit; GE Healthcare, Buckinghamshire, UK).
